# Aberrantly hypermethylated Homeobox A2 derepresses metalloproteinase-9 through TBP and promotes invasion in Nasopharyngeal carcinoma

**DOI:** 10.18632/oncotarget.1367

**Published:** 2013-11-04

**Authors:** Hsin-Pai Li, Chen-Ching Peng, I-Che Chung, Mei-Yuan Huang, Shao-Tung Huang, Chia-Chun Chen, Kai-Ping Chang, Cheng-Lung Hsu, Yu-Sun Chang

**Affiliations:** ^1^ Graduate Institute of Biomedical Sciences, Chang Gung University, Taoyuan, Taiwan, Republic of China (ROC); ^2^ Molecular Medicine Research Center, Chang Gung University, Taoyuan, Taiwan, Republic of China (ROC); ^3^ Department of Microbiology and Immunology Medical School, Chang Gung University, Taoyuan, Taiwan, Republic of China (ROC); ^4^ Department of Otolaryngology-Head and Neck Surgery, Chang Gung Memorial Hospital, Lin-Kou, Taiwan, ROC; ^5^ Division of Hematology-Oncology, Chang Gung Memorial Hospital, Lin-Kou, Taiwan, ROC

**Keywords:** HOXA2, DNA methylation, NPC, MMP9

## Abstract

Nasopharyngeal carcinoma (NPC) is notorious for its high invasiveness and metastatic ability. In this study, we identified a differential hypermethylated transcription repressor, *Homeobox A2 (HOXA2)*, which may render NPC cells invasive and metastatic. Aberrant hypermethylation of *HOXA2* led to low RNA expression in NPC tumors and cells. Addition of methylation inhibitor 5'Aza restored *HOXA2* RNA expression in NPC cells. Methylated *HOXA2* promoter reduces the binding affinity of the transcriptional co-activator p300, causing transcriptional repression of *HOXA2*. In NPC cells, re-expression of ectopic HOXA2 was correlated with decreased invasive ability and reduced metalloproteinase *MMP-9* RNA and protein expression. Promoter, ChIP and DNA-pull down assays indicated that HOXA2 competes with the transcription activator, TATA-box binding protein (TBP) for a recognition sequence near the *MMP-9* transcription start site, and suppresses *MMP-9* transcription. Thus, HOXA2 acts as a suppressor or TBP-antagonist to inhibit *MMP-9* expression; while methylation-mediated inactivation of *HOXA2* in NPC derepresses MMP-9 production and increases invasion of NPC cells. In NPC plasma samples, increased plasma EBV copy number was correlated with increased in cell-free *HOXA2* hypermethylation and elevated MMP-9 levels. Plasma EBV DNA and methylated cell-free HOXA2 can be used as biomarkers for monitoring NPC treatment.

## INTRODUCTION

Nasopharyngeal carcinoma (NPC) is a common head and neck cancer in Southeast Asia [1]. Diet, genetic background and Epstein-Barr virus (EBV) type II latent infection are strongly associated with NPC [2, 3]. A viral oncoprotein expressed during latent EBV infection, latent membrane protein 1 (LMP1), is considered to be a key factor in NPC development [4]. Previously, we demonstrated that LMP1 activates DNA methyltransferase 1 (DNMT1) and causes aberrant cellular DNA hypermethylation, leading to inactivation of the adhesion molecule, E-cadherin [5, 6]. This indicates that the viral protein can silence gene via hypermethylation and promote NPC tumor progression. DNA methylation is essential for normal development, gene imprinting and X chromosome inactivation [7, 8]. However, aberrant DNA methylation has been associated with tumor suppressor gene (TSG) inactivation and tumorigenesis in many cancers [9]. We characterized differentially hypermethylated genes in NPC tumors and identified that the *Homeobox A2 (HOXA2)* gene is hypermethylated and down-regulated at the RNA level. There are 39 *HOX* genes comprising four different chromosomal clusters A~D in human. The HOX proteins are highly conserved transcription factors that share a similar DNA-binding domain called the homeodomain [10]. The *HOX* genes are conserved among species and are important for body segment patterning [11, 12]. HOXA2 protein may suppress gene expression through the HOXA2-response element [13, 14]. The biological significance of *HOXA2* hypermethylation in NPC cells will be discussed.

## RESULTS

### Differentially methylated HOXA2 correlates with low mRNA expression in NPC biopsies and cell lines

To identify genes that were differentially methylated in NPC, we isolated genomic DNA from 4 tumor and non-tumor (T-N) paired NPC biopsies and analyzed the samples on TranSignal Methylation Promoter Arrays (Panomics). Among the tested 82 promoters, *HOXA2* was found to be hypermethylated in NPC compared with normal adjacent tissues. In three of the four cases, the *HOXA2* promoter hybridization signal in NPC tumors was stronger than that in the non-tumor counterparts (Fig. S1; left panel), indicating the amount of methylated *HOXA2* DNA was comparatively higher in tumors. In contrast, the promoter hybridization signal for the *IRF7* gene (as negative control) was similar between tumor and non-tumor samples (Fig. S1; right panel).

**Figure 1:**
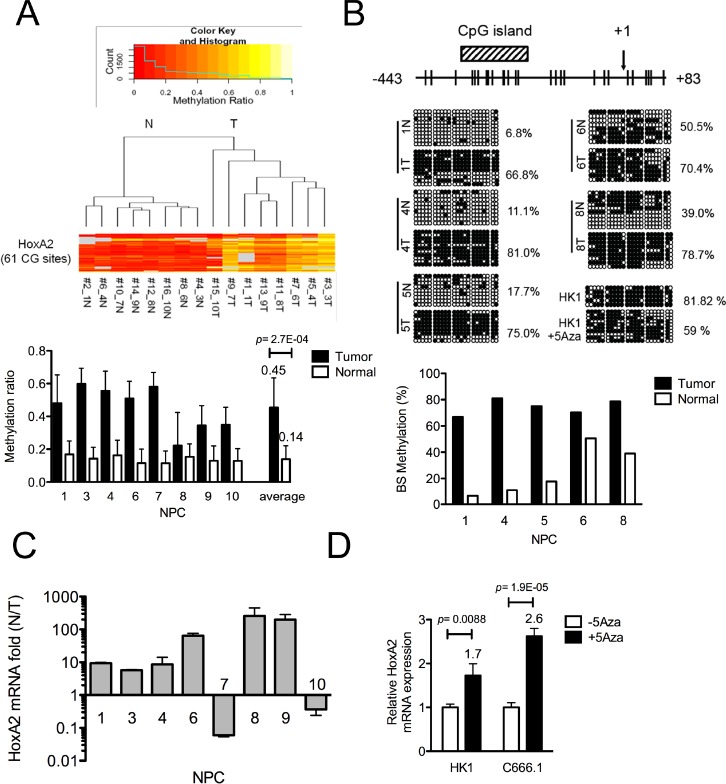
Identification of the HOXA2 gene as differentially methylated in NPC (A) Three fragments covering the 61 CpG sites in the *HOXA2* promoter (−1492~+17) were amplified from 8 paired NPC samples and analyzed by MassArray (Sequenom®). The colored histogram represents the CpG methylation ratio, ranging from 0 (0% methylated CpG site, red) to 1.0 (100% methylated CpG site, yellow). Each horizontal lane denotes the methylation ratio of a given CpG site in NPC tumor and adjacent normal tissues. The right panel shows the averaged methylation ratio of each sample. (B) Schematic map containing a CpG island (−345~−205, diagonal region) of the *HOXA2* promoter, as adapted from the MethPrimer website [15]. The transcription start site (+1) and 22 CpG sites (vertical bars) are indicated. Bisulfite sequencing analysis was performed on −443~+83 in 5 paired NPC clinical samples. Each horizontal row represents a single clone; the methylation percentages of at least 8 individual clones are indicated, with unmethylated (○) and methylated (●) CpG sites. The lower panel shows the methylation percentage for each sample. (C) Q-RT-PCR analysis of *HOXA2* mRNA expression was performed on the 8-paired NPC samples, and the results were normalized with respect to *GAPDH* expression. The columns represent the relative *HOXA2* mRNA expression fold-change in log value (N/T). (D) Columns represent the Q-RT-PCR data of relative fold-change of restored *HOXA2* mRNA expression normalized with respect to *GAPDH* expression in NPC cell lines (HK1, C666.1) (+/−) 10μM 5'Aza treatment.

To confirm that the *HOXA2* promoter is hypermethylated in NPC, we used MassARRAY mass spectrometry (Sequenom) to quantify the *HOXA2* methylation status in 8 T-N paired NPC biopsies (Supplementary MassARRAY methylation ratio). The MassARRAY methylation profile heat map for the 8 T-N paired NPC samples comprised 61 CpG units (−1421~+17) (Fig. 1A; upper panel). NPC tumors with high methylation ratios or non-tumor tissues with low methylation ratios grouped separately in unsupervised clustering. The overall methylation ratio (the average of all tumors or non-tumors) for the 8 NPC tumors was 0.45, whereas that of the adjacent normal counterparts was 0.14 (Fig. 1A; lower panel). Therefore, the overall fold-change of the average methylation ratios of the tumor and non-tumor samples was 3.3 (***, p < 0.001), suggesting that the *HOXA2* promoter is hypermethylated in NPC tumors.

To identify which CpG sites of the *HOXA2* promoter are differentially methylated in NPC samples and cell lines, we performed bisulfite sequencing. MethPrimer web-based prediction [15] indicated that there was one CpG island (−345~−205) located near the transcription start site (TSS, +1) of *HOXA2* promoter region (Fig. 1B). Our bisulfite sequencing results indicated that −443~+83 of which contained the CpG island was hypermethylated in the NPC cells (85.8% in HK1 and 94.8% in C666.1). As shown in Fig. 1B, the methylation percentage of the same *HOXA2* region was high in five clinical tumor samples (66.8~81%, average 74%) compared with their adjacent normal tissues (6.8~50.5%, average 25%).

Based on this differentially methylated region (DMR), we designed methylation-specific *HOXA2* primers to quantify the methylation percentages of the same NPC clinical samples by quantitative methylation specific PCR (Q-MSP-PCR) analysis. As shown in Fig. S2, strong MSP-PCR products can be detected in all NPC tumors and a weak PCR product in one of the non-tumor samples, indicating again the methylation percentage in NPC tumors was higher than that of non-tumors. The quantitative values of Q-MSP methylation percentage of paired NPC tumor (T) and non-tumor (N) tissues were indicated in Fig. S3A, (T: 31.2~87.9%, average 50.9%; N: 1.8~28.1%, average 6.7%). Thus, the results from three different methylation-detecting assays all indicated that *HOXA2* was differentially hypermethylated in NPC tumor tissues.

We then used quantitative (Q)-RT-PCR to evaluate the *HOXA2* mRNA levels in the same NPC tumor tissues, and found that generally *HOXA2* mRNA levels were reduced 5-~210-fold (compared with the corresponding adjacent normal tissues) in six out of the eight NPC tumors (Fig. 1C). The relative quantitative values of *HOXA2* mRNA level of paired NPC tissues were indicated in Fig. S3B. Our data also indicated that the *HOXA2* DNA methylation level (Q-MSP methylation %) was negatively correlated with the relative RNA expression levels [−ΔCt= −(Ct_*HOXA2*_−Ct_*GAPDH*_)] in NPC samples (Fig. S3C; correlation r = −0.45). Furthermore, the *HOXA2* mRNA levels in the two NPC cells, C666.1 and HK1, could be restored by addition of the demethylation agent, 5'Aza (Fig. 1D), suggesting that DNA methylation was responsible for the transcriptional repression of *HOXA2* in NPC cells.

### DNA methylation suppresses HOXA2 promoter activity by impairing the binding of the transcription activator, p300

To determine whether DNA methylation *per se* can silence *HOXA2* promoter activity, we performed patch methylation assays [16]. The *HOXA2* promoter was PCR-generated and *in vitro* methylated by the methylase, SssI. Methylation was verified by digestion with the methylation-sensitive restriction enzyme, BstUI (Fig. S4A). Methylated (p*HOXA2*^me^) and unmethylated (p*HOXA2*^un^) promoters were inserted into a luciferase reporter and separately transfected into 293T cells. The results indicated that the p*HOXA2*^me^ had only 33.4% of the p*HOXA2^un^* promoter activity (Fig. S4B). This *in vitro* result supports our observation in clinical NPC samples that DNA methylation significantly suppresses *HOXA2* promoter activity.

**Figure 2:**
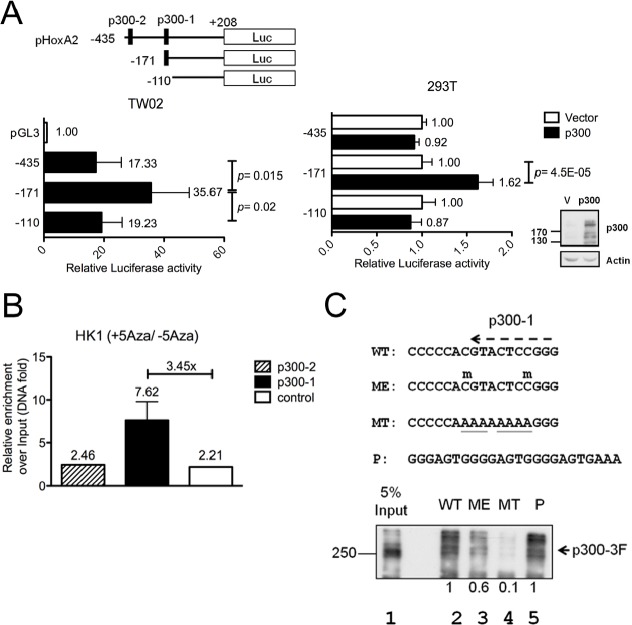
Methylation inhibits HOXA2 promoter activity by impairing binding of the transcription activator, p300 (A)Three HOXA2 promoter deletion clones and two putative p300 binding sites (p300-1 and p300-2) are indicated. Different *HOXA2* promoter deletion clones were transfected into TW02 cells (left panel) or different *HOXA2* promoter deletion clones and the pCMV/p300 expression clone were co-transfected into 293T cells (right panel). Relative luciferase activities were detected 48hr post-transfection and all the luciferase reporter activities were normalized with respect to renilla activity. The expression levels of p300 and actin (internal control) were examined by Western blotting. (B) ChIP assays were performed with anti-p300 antibody or isotype IgG, using HK1 nuclear extracts treated with or without 10μM 5'Aza. Q-PCR was used to amplify p300-1 (−171~−55), p300-2 (−435~−254), and a downstream region lacking a p300 binding site (−110~+208). (C) DNA pull-down was used to analyze the binding affinity of exogenous Flag-tagged p300 (p300-3F) to wild-type (WT), methylated (ME), mutated (MT) *HOXA2* p300-1 and a three copies of p300 consensus (P) biotinylated probes. Anti-Flag antibody was used to examine the amount of bound p300-3F in the immunoprecipitates and 5% inputs. Methylated cytosine is indicated by “m” above C, and mutated sequences were underlined.

To test whether methylated CpG sites impair the binding of transcription factors, we used TFSEARCH to analyze transcription-factor-binding motifs in the *HOXA2* promoter, focusing on the DMR identified by our bisulfite sequencing analysis. One of the selected transcription factors was p300, a transcriptional co-activator that functions as a histone acetyltransferase and regulates chromatin remodeling [17]. Two putative p300 binding sites close to the TSS were identified in the *HOXA2* promoter: p300-1 (−137) and p300-2 (−290). To examine whether these p300 sites were crucial for *HOXA2* promoter activation, p*HOXA2-*Luc deletion reporters were generated (Fig. 2A, upper panel) and transfected into TW02 cells (have high-level endogenous p300 expression). Higher relative luciferase activity was detected from pHOXA2(−171) 35.67-fold versus p*HOXA2*(−435) 17.33-fold, and p*HOXA2*(−110) 19.23-fold (Fig. 2A, left panel), suggesting that p300-1 is important for *HOXA2* promoter activity. Similar results were obtained when a p300 expression plasmid was co-transfected into 293T cells (have low-level endogenous p300) along with the same p*HOXA2*-Luc deletion reporters. The promoter activity of p*HOXA2*(−171) was further activated 1.62-fold in the presence of p300 (Fig. 2A, lower panel), while the activities of the two other promoters were unaltered in the presence of p300. Thus, our promoter assays indicate that p300 activates the *HOXA2* promoter mainly via the p300-1 site.

To confirm whether the p300-binding affinity of the *HOXA* promoter could be affected by DNA methylation, we performed quantitative ChIP assays using p300 antibody and HK1 cells with or without 5'Aza treatment. Our data showed that DNA demethylating agent, 5'Aza, enhanced the binding of p300 to the p300-1 site (by 3.45-fold) but not the p300-2 site (1.1-fold) on the *HOXA2* promoter when compared with the untreated control cells (Fig. 2B). These results indicate that p300 binds to the p300-1 site but not to the p300-2 site.

Next, we used DNA pull-down experiments to test whether DNA methylation of two CpG sites on *HOXA2* p300-1 site could affect the p300-binding affinity. We synthesized double-stranded biotinylated oligomers containing the p300-1 site with unmethylated (WT) or methylated (ME) CpG sites; mutated p300 sequence (MT) as negative control; and three copies of p300 consensus sequence (P) as positive control. Biotinylated probes were bound to nuclear extracts overexpressing Flag-tagged p300 (p300-3F), precipitated by streptavidin beads followed by Western blotting. The results showed that p300-3F had a relative strong binding with the WT probe (Fig. 2C, lane 2, considered as 1) and the positive control (lane 5) but had a weak binding with the ME probe (lane 3, 0.6-fold). However, the binding of p300 was abolished towards the mutant MT probe (lane 4). These results indicate that p300 has a lower binding affinity for the methylated p300-1 binding site of the *HOXA2* proximal promoter, resulting in lower *HOXA2* expression. Conversely, demethylation of the *HOXA2* promoter by 5'Aza appears to promote binding of p300 to the p300-1 site and restore *HOXA2* expression.

### Functional analysis of HOXA2-expressing NPC cell lines

**Figure 3:**
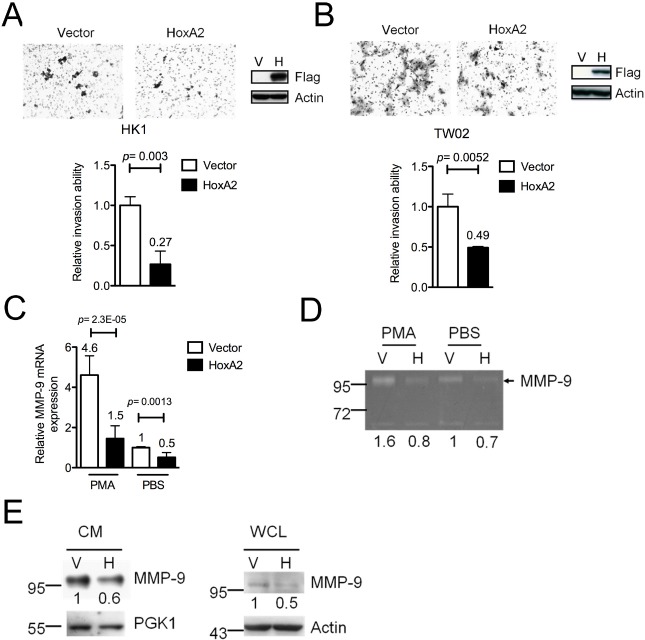
HOXA2 inhibits cell invasion by down-regulating MMP-9 expression in NPC cells The cell invasion abilities of (A) HK1 and (B) TW02 cells stably expressing HOXA2 and vector were analyzed. Migrated cells were counted from 10 different microscopic fields, and Western blot analysis was used to confirm the expression of exogenous HOXA2 (anti-Flag) and the internal control (anti-actin). (C) *MMP-9* mRNA, (D) MMP-9 enzymatic avtivities and (E) MMP-9 protein in cultured medium (CM) and whole cell lysate (WCL) of vector control (V) and HOXA2-expressing (H) HK1 cells were measured by QRT-PCR, gelatin zymography and Western blotting. Cells were either treated with or without PMA. PGK1 and actin were the protein loading control in CM and WCL, respectively. (A-E) Three independent experiments were performed in duplicate, and the results are shown as mean±SD (* p<0.05, ** p<0.01, and *** p<0.001).

Silencing of TSGs via promoter hypermethylation is an early event in various cancers [7]. If inactivation of *HOXA2* contributes to NPC formation, then re-expression of *HOXA2* in NPC cells might reverse the oncogenic features of NPC cells. A recombinant lentivirus expression system was used to stably overexpress HOXA2 or the vector control in HK1 and TW02 cells. Proliferation, colony formation and cell invasion assays were used to examine the biological functions of HOXA2 *in vitro*. No significant difference was observed in the growth rate and colony formation of HOXA2-expressing HK1 (Fig. S5A) or TW02 cells (Fig. S5B) compared to control cells. However, cell invasion assays indicated that fewer HOXA2-expressing HK1 cells (0.27-fold) and TW02 cells (0.49-fold) invaded across the membrane compared with control cells (Fig. 3A) demonstrating that HOXA2 expression inhibits the cell invasion ability. And this inhibition was not due to a difference in cell proliferation between HOXA2-overexpressing and control cells.

### HOXA2 down-regulates MMP-9 expression and enzymatic activity

Previous reports indicated that cell invasion ability can be promoted by overexpression of matrix metalloproteinases (MMPs), which degrade various extracellular components [18]. Next, we analyzed 22 *MMPs* expression from the cDNA microarray (Affymetrix) data of nine NPC tumors versus one combined adjacent normal (control). We found that 8 out of 22 MMPs were overexpressed (>1.5-fold) in NPC tumors when compared with the control (Table S3). The top of the MMPs list was MMP-9 (40-fold higher in NPC tumors compared with control), one of the best-studied proteinases involved in promoting tumor invasion and metastasis. In fact, MMP-9 has been reported to facilitate NPC tumorigenesis in many studies [19-21]. As HOXA2 reportedly functions as a transcriptional repressor during development [13, 22], we hypothesized that HOXA2 might repress MMP-9 expression. We examined the mRNA, protein expression level and enzymatic activity of MMP-9 in HOXA2-expressing and control NPC cells. As expected, *MMP-9* mRNA levels were significantly reduced (to ~50%) in HOXA2-expressing HK1 cells compared with control cells (Fig. 3C). Even in the presence of phorbol myristate acetate (PMA), a known chemical that activates MMP-9 expression [23], the mRNA level of *MMP-9* was again significantly lower in HOXA2-expressing HK1 cells (1.45-fold) compared with control cells (4.60-fold). Western blotting showed that in the presence of HOXA2 the protein level of MMP-9 was downregulated in both cultured medium and whole cell lysates (Fig. 3E). Gelatin zymography assays indicated that HOXA2 expression moderately suppressed the enzymatic ability of MMP-9 (0.73-fold) with respect to its activity in control cells (Fig. 3D, compare lane 3 and 4). In PMA-treated cells, HOXA2 overexpression was associated with a ~50% reduction in MMP-9 enzymatic activity (0.82-fold) compared with control cells (1.56-fold) (Fig. 3D, compare lane 1 and 2). Collectively, HOXA2 represses *MMP-9* RNA and protein expression in NPC cells.

### HOXA2 antagonizes TBP by binding to TATA-box and suppresses MMP-9 transcription

**Figure 4:**
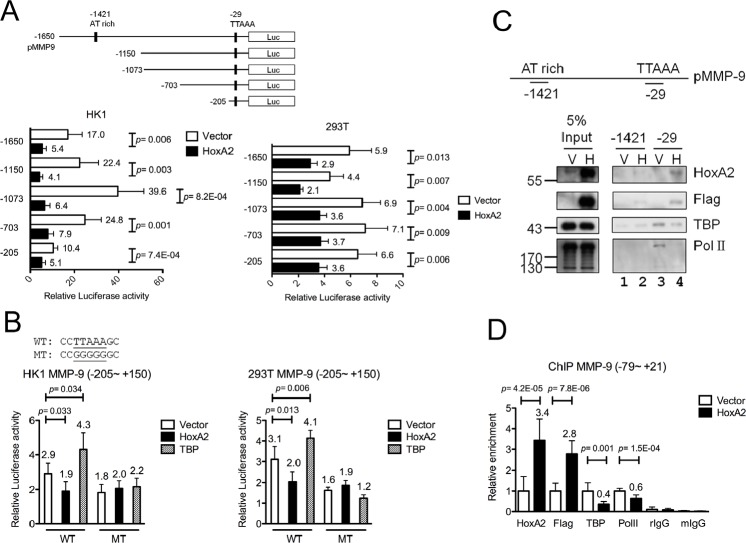
Direct binding of HOXA2 to the MMP-9 TATA box region interferes with the TBP-mediated activation of MMP-9 (A) MMP-9 promoter deletion clones and the pCMV/HOXA2 expression vector or (B) mutated promoter MT (−205~+150) and pCMV/HOXA2 or pCMV/TBP were co-transfected into HK1 and 293T cells, and luciferase reporter activity was detected. (C) In vitro binding of HOXA2, TBP and RNA PolII on the MMP-9 promoter was determined by DNA pull-down assays. Biotinylated probes containing the MMP-9 TATA box (−39~−15) or an upstream AT-rich region (−1421~−1397) were incubated with nuclear extracts from HK1 cells stably expressing vector or HOXA2. Western blotting was used to examine the expression of HOXA2, Flag-tagged HOXA2, TBP and PolII in the immunoprecipitates and 5% inputs. (D) ChIP assays were performed with antibodies against HOXA2, Flag, TBP, PolII, or isotype IgGs using nuclear extracts from HK1-expressing or control cells. Q-PCR was used to amplify the MMP-9 promoter TATA box (−3~−15). (A-E) The presented data reflect three independent experiments, and the results are shown as mean±SD (* p<0.05, ** p<0.01, and *** p<0.001).

To investigate whether HOXA2 is a transcriptional repressor of *MMP-9*, we generated various *MMP-9* promoter deletion clones containing potential HOXA2-binding sites. HOXA2 binds to the TAAT target sequence to down-regulate the transcription of its target genes [14, 24, 25]. A putative HOXA2-response element was identified at the *MMP-9* promoter (−1421). We constructed five *MMP-9* promoter Luc reporters (−1650, −1150, −1073, −703 and −205~+150; Fig. 4A). *MMP-9* promoter clones and HOXA2-expressing or control vectors were co-transfected into HK1 and 293T cells. Luciferase assays revealed that the promoter activities of all five *MMP-9* promoter deletion clones, including the shortest −205~+150, were suppressed in HOXA2-overexpressing cells compared with vector control (Fig. 4A). Sequence analysis revealed that the only possible HOXA2 binding site within the (−205~+150) *MMP-9* promoter was the TATA-like sequence, TTAAA (−29), next to the TSS. Luciferase assays indicated that co-transfection of either HOXA2 or TBP expression clones with the shortest *MMP-9* promoter reporter could either suppress slightly or activate the promoter activities (Fig. 4B). To test whether the TATA-like sequence was responsible for the transcriptional repression or activation, by HOXA2 or TATA-binding protein (TBP), respectively, we generated a site-directed *MMP-9* promoter mutant, denoted as −205 MT, in which the TTAAA sequence was mutated to GGGGG. Luciferase assays demonstrated that this mutation abolished the HOXA2-mediated repression or TBP-mediated activation of *MMP-9* transcription when compared to wild type reporter (Fig. 4B) suggesting that the TATA-like sequence is crucial for the recognition of both HOXA2 and TBP.

To further clarify the HOXA2-binding sites in the *MMP-9* promoter, we performed DNA pull-down experiments using the two double-stranded biotinylated oligonucleotide probes containing the putative HOXA2-response element (−1421) and the TATA-like sequence (−29), respectively. Biotinylated probes were bound to nuclear extracts of vector- or HOXA2-expressing HK1 cells, purified by streptavidin beads followed by Western blotting. The results revealed that HOXA2 had a relatively strong affinity toward TATA-like sequence (−29) in HOXA2-expressing HK1 extracts (Fig. 4C; anti-HOXA2, anti-Flag, lane 4) when compared with vector control extracts (Fig. 4C, lane 3). Little or no HOXA2 binding was observed to the putative HOXA2-response element (−1421) in vector- or HOXA2-expressing HK1 extracts (Fig. 4C, lanes 1 and 2) indicating that HOXA2 binds to the *MMP-9* promoter at (−29) but not (−1421). Based on these findings, we hypothesized that HOXA2 may compete with TBP to block the binding of RNA polymerase II (RNA Polll). Additional Western blot analyses using the same DNA pull-down lysates showed that significant TBP and RNA PolII bound to the TATA-like sequence (−29) probes (Fig. 4C; anti-TBP and anti-RNA PolII, lane 3) in the absence of HOXA2 expression, but both bound only weakly in the HOXA2-expressing HK1 extracts (Fig. 4C, lane 4). This suggests that the TBP and HOXA2 factors may compete for the same binding site at (−29). The presence of HOXA2 could interfere or compete with the binding of TBP and RNA PolII to the *MMP-9* promoter.

To address whether HOXA2 and TBP can directly bind to the *MMP-9* TATA-like sequence, we used purified recombinant His-tagged HOXA2 and TBP proteins to perform EMSAs. We found that recombinant HOXA2 and TBP proteins could both bind directly to the *MMP-9* TATA box biotin-labeled probe (Fig. S6, solid arrows, lane 2 and 5). Furthermore, the two proteins might form higher-molecular-weight dimers or multimers with the probes (Fig. S4, dashed arrows, lane 2 and 5). Incubation with the corresponding antibodies led to the formation of a supershifted complex and a marked decrease in the intensity of the lower band (Fig. S6, asterisks, lane 3 and 6). However, when the TTAAA core sequence was mutated, the binding affinity between the recombinant HOXA2 or TBP proteins and mutant probe dropped dramatically (Fig. S6, lane 11 and 12).

To test whether HOXA2 could interfere with TBP binding and inhibit *MMP-9* transcription *in vivo*, we performed quantitative ChIP assays on the *MMP-9* promoter in HOXA2-expressing and vector control cells. In HOXA2-expressing cells, HOXA2 bound to the *MMP-9* TATA box region and reduced the binding of TBP and RNA PolII to the same region (Fig. 4D), compared with the binding in vector control cells. These results indicate that HOXA2 may antagonize TBP binding and RNA PolII recruitment to the *MMP-9* TATA box region, thereby decreasing *MMP-9* transcription.

### HOXA2 methylation status positively correlates with EBV copy number and MMP-9 levels in plasma from NPC patients

**Figure 5:**
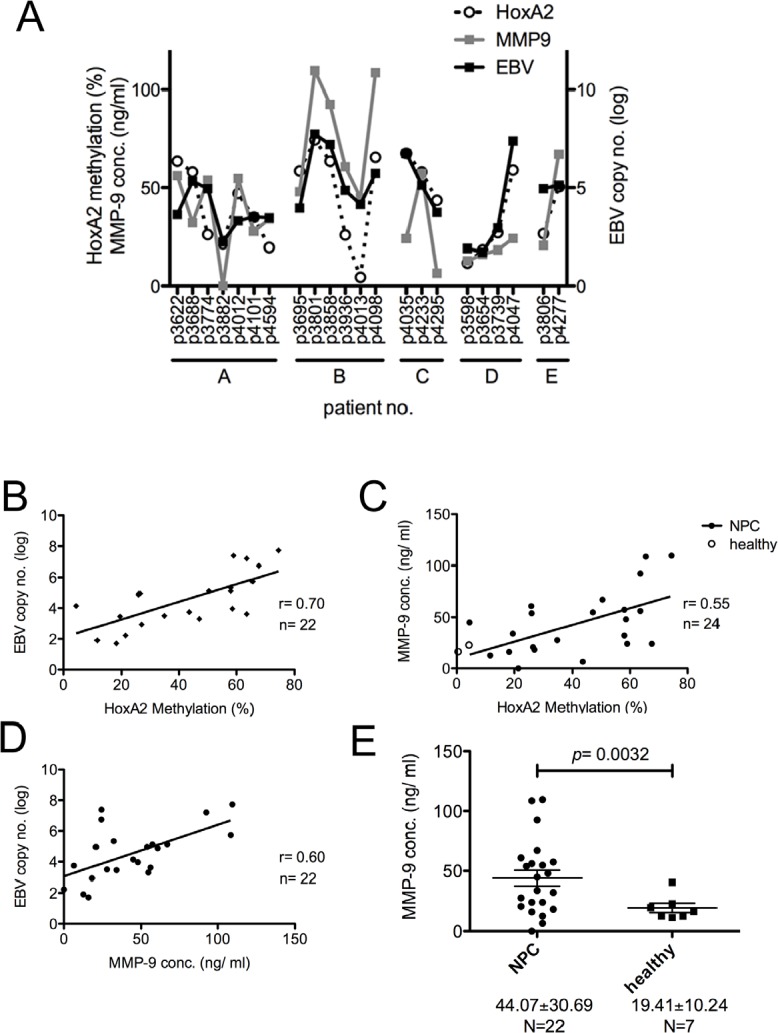
Correlation of EBV copy number, HOXA2 methylation status and MMP-9 concentration in plasma samples from NPC patients Plasma was collected from five NPC patients at different time intervals (1~3 months) after standard NPC treatments (including radiation and chemotherapy) were administered according to the CGMH guidelines (N=22). (A-D) Correlations were observed between the log EBV DNA copy number, *HOXA2* methylation status and plasma MMP-9 concentration. Pearson's correlation coefficients (r) are indicated. (E) Plasma MMP-9 concentrations from 22 NPC samples and seven healthy donors were measured by ELISA. The results are shown as mean±SD (** p<0.01).

It is generally accepted that the cell-free DNA (cfDNA) content is higher in cancer patients compared with healthy people [26]. To test whether the levels of (i) EBV copy number, (ii) *HOXA2* methylation ratio and (iii) MMP-9 levels are inter-correlated in a given sample, we obtained 3~5 follow-up plasma samples from five treated NPC patients (Fig. 5, designated A~E; N=22), and EBV copy number in the plasma was monitored by q-PCR; and cfDNA was extracted from the samples and methylation specific-high resolution melting (MS-HRM) (Fig. S7 and Table S4) and q-PCR was performed to detect the *HOXA2* methylation ratio; and the MMP-9 levels of the plasma samples were detected by the ELISA. Moderate to strong positive correlations were observed when we compared the levels of any two of the three factors (Fig. 5, r=0.55~0.7) suggesting EBV titer, *HOXA2* methylation and MMP9 level are inter-correlated with each other. Interestingly, the values of the three factors exhibit similar trends at a given time in a patient; however, the values vary at different time reflecting the clinical outcome or tumor burden of a patient during therapy. It appears that the higher the EBV copy number, the higher the *HOXA2* methylation status and the higher the MMP-9 in the plasma of NPC patients.

## DISCUSSION

We herein identify two dysregulated transcription cascades that contribute to NPC tumorigenesis: (1) DNA methylation of *HOXA2* impairs the binding of transcription activator p300 on the *HOXA2* promoter; (2) the inactivation of *HOXA2* promotes TBP binding on *MMP-9* promoter and activates the *MMP-9* expression. Our data indicated that methylation of *HOXA2* at the proximal p300-1 site interfered p300 binding, thereby suppressing *HOXA2* expression. Although the core consensus sequence for p300 in the *HOXA2* promoter (NNNG/AGGAGTNNNC/G) does not contain an obvious CG site; however, two CG sites were found flanking the central p300-1 sequence. Thus, methylation of flanking sequences could interfere with protein binding via steric hindrance.

The *HOXA2*, located within *HOXA* gene cluster on human chromosome 7, is important for neural-crest-derived structures development and the segmentation of neuromeres in hindbrain [27, 28]. HOXA2 protein acts as a transcription regulator, and may suppress gene expression through the HOXA2-response element [13, 14]. Through direct binding to TAAT consensus sequence, HOX proteins may regulate the transcription of their target genes and thereby control cell differentiation during development [10, 29]. Dysregulation of *HOXA2* may have negative impacts on cells and/or lead to oncogenesis [30]. Aberrant expressions of *HOX* genes have been correlated with breast cancer development and the malignant behavior of cancer cells [31]. Furthermore, the *HOXA* and *HOXD* clusters are hotspots for *de novo* methylation of CpG islands in human lung adenocarcinomas [32]. Temporal and spatial changes in *HOX* expression in developing and normal cells are tightly controlled. It would be interesting to test if other *HOX* members are transcriptionally repressed by DNA methylation and/or functionally antagonized by TBP.

This is the first report to demonstrate HOXA2 can compete with the TBP for the TATA-box due to their DNA consensus sequence resemblance. Thereby, TBP and HOXA2 can co-regulate the *MMP-9* transcription but they act in an opposite fashion. Conceivably, in addition to *MMP-9*, other HOXA2-TBP-targeted genes' transcription may also be affected in NPC. The MMP family has 29 members; among these members MMP-2 and MMP-9 were found to have increased enzymatic activity and associations with metastasis in several cancers [18, 33] including NPC [21]. Two previous reports indicated that the MMP-9 overexpression observed in NPC was correlated with the EBV LMP1-mediated NF-κB signaling pathway [34] and the EBV Zta-mediated AP1 pathway [35]. Furthermore, increased MMP-9 expression in NPC tumors has been correlated with poor prognosis [36]. Our present findings offer an alternative explanation for MMP-9 overexpression in NPC.

While normal healthy individuals have little cfDNA in their plasma (~30 ng/ml), a high level of circulating cfDNA (~180 ng/ml) can be detected in the plasma of cancer patients [26]. This difference reflects that cfDNA is mainly released from apoptotic and necrotic tumor cells; and the amount of cfDNA may be used as a cancer indicator [37]. As it is easier to obtain plasma samples than biopsies, measurement of aberrant methylated cfDNA in plasma may replace biopsies in NPC patients. Furthermore, the sensitivity and accuracy of MS-HRM is comparable to that of bisulfite sequencing suggesting that MS-HRM could be used as a rapid, sensitive and economical method for assessing *HOXA2* methylation in both tumors and plasma samples of NPC patients. The 22 plasma samples collected from 5 NPC patients at different time intervals showed that EBV copy number, cfDNA *HOXA2* methylation percentage and the level of MMP-9 are inter-correlated. The dynamic levels of these three parameters vary over time within the same NPC patient and they may all serve as plasma biomarkers reflecting the tumor load of both *in-situ* and metastatic NPC tumors.

**Figure 6:**
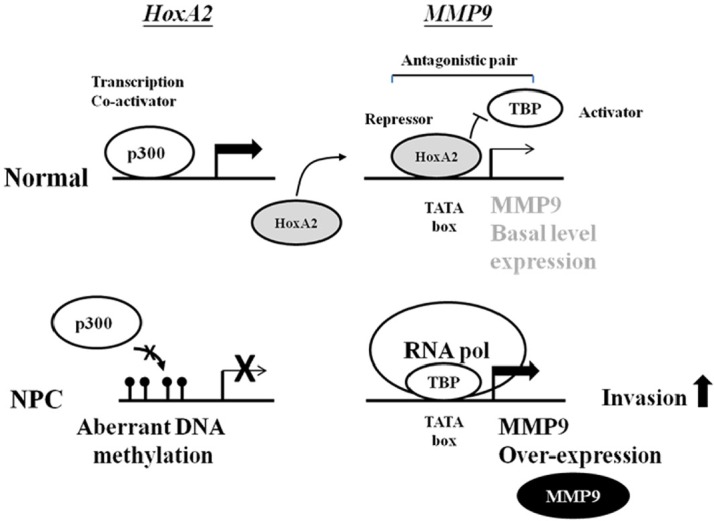
Schematic model of silencing of the transcription repressor HOXA2 by aberrant hypermethylation enhances invasion via MMP-9 activation in NPC In normal cells, transcription of *HOXA2* is activated by p300 binding. Subsequently, HOXA2 binds to the *MMP-9* TATA-box and interferes the binding of TBP resulting in suppression of *MMP-9* expression. In NPC cells, methylation of the *HOXA2* impairs the p300 binding thereby inactivates the *HOXA2* gene. In the absence of transcription repressor HOXA2, TBP and RNA polII can bind to the TATA-box of *MMP-9* and activate *MMP-9* expression. Elevated MMP-9 level, in turn, promotes the invasiveness of NPC cells.

Finally, we propose a model in which illustrates aberrant hypermethylation of *HOXA2* increases MMP-9 expression and promotes the invasiveness of NPC cells (Fig. 6). Silencing *HOXA2* through DNA methylation may have profound effect on gene expression profile in NPC when considering there are ~24% of the human genes which contain TATA-like element [38]. The final outcome for cells with *HOXA2* impairment may therefore depend on the overall functions of all the downstream targets of HOXA2 and/or HOXA2-TBP.

## MATERIALS AND METHODS

### Cell lines, NPC tissue samples and plasma

The NPC cell lines, C666.1 [39] and HK1 [40], were provided by Dr. S. W. Tsao (Hong Kong University, SAR, China), and were cultured in 10% fetal bovine serum (FBS; Gibco)/RPMI1640 (Gibco BRL, Rockville, MD). The TW02 cell line was obtained from Dr. C. T. Lin (National Taiwan University, Taiwan); TW02 and the embryonic kidney cell line, 293T, were cultured in 10% FBS/DMEM (Gibco). C666.1 and HK1 cells were treated with 10 μM demethylation agent 5' Aza for 5 days, with daily replacement of fresh medium. Eight pairs of frozen NPC tumor and adjacent normal biopsies (< 2 mm) were collected from Chang Gung Memorial Hospital (CGMH; Taiwan) by Dr. K. P. Chang. Genomic DNA and RNA of NPC biopsies were simultaneously extracted using the TRIZOL reagent (Invitrogen, Carlsbad, CA). Plasma was collected from five NPC patients at CGMH by Dr. C. L. Hsu. Plasma cell-free DNA was extracted by using QIAamp DNA kit (Qiagen, Germantown, MD). This study was reviewed and approved by the IRB and ethics committee of CGMH (IRB:97-1226A3 and IRB:95-0692B).

### Quantification of DNA methylation in NPC biopsies by MassArray MALDI-TOF MS analysis

Genomic DNA (2 μg) from all 16 tissue samples were bisulfite treated using the EZ DNA Methylation Kit (Zymo Research, Irvine, CA) and subjected to PCR amplification using three sets of *HOXA2*-specific bisulfite PCR primers. The extent of methylation at the individual CpG sites of these PCR products was further analyzed by Sequenom MassArray Epityper (Sequenom, San Diego CA) [41]. The degree of methylation for each CpG site was determined using the ratio between methylated-C and total cytosine, which ranged from 0 to 1.

### Bisulfite sequencing

Bisulfite-converted DNA (100 ng) was amplified using specific bisulfite sequencing (BS) primers that were designed using Methprimer (http://www.urogene.org/methprimer/, UCSF). The resulting PCR products were cloned, and 8~10 individual clones were sequenced.

### Cell proliferation and invasion assay

Cell proliferation was determined by trypan blue (Gibco) exclusion and cell counting. Stable HOXA2-expressing and vector control HK1 (2×10^5^) or TW04 (5×10^4^) cells were seeded in 6-well plates, and cell numbers were counted daily for 5 days. Cell invasion was assessed using a cell invasion assay kit (Chemicon, Temecula, CA). Briefly, HOXA2-expressing HK1 (5×10^5^) or TW02 cells (4×10^5^) were seeded to the upper chamber and cultured for 24 hr (TW02) or 72 hr (HK1), allowing cells to migrate through the 0.8−μm membrane. The membrane was then excised and stained with 0.1% crystal violet; and cells were counted under a microscope. All experiments were repeated at least three times, each in duplicate.

### Promoter analysis and luciferase assay

The *HOXA2* and MMP-9 luciferase reporters, pHOXA2/pGL3 and pMMP-9/pGL3, were amplified and both subcloned into the *Bgl*II and *Hin*dIII sites of the pGL3-basic vector (Promega, Madison, WI). To normalize each transfection reaction, a renilla reporter plasmid (pRL-CMV; Promega) (one-tenth of the total DNA amount) was co-transfected with the luciferase reporters. Cells were harvested 48 hr post-transfection and lysed, and promoter activity was assayed using the Dual Luciferase Assay

System (Promega) on a GLOMAX 20/20 Luminometer (Promega).

### Antibodies for Western blot analyses

Primary antibodies: anti-Flag antibody (M2; Sigma), p300 antibody (Santa Cruz Biotech, Santa Cruz, CA), and anti-actin monoclonal antibody (MDBio, Taipei, Taiwan) and HRP-conjugated secondary antibodies (goat anti-mouse antibody and goat anti-rabbit antibody) were used. The results were visualized using an ECL detection kit (Millipore, Billerica, MA).

### Quantitative chromatin immunoprecipitation (ChIP) assay

ChIP assays were performed *as previously described [42]*. Either 1 μg of anti-HOXA2 antibody or 2 μg of the other specific antibodies (anti-p300, Santa Cruz; Flag® M2, Sigma; anti-TBP, Santa Cruz; anti-RNA polymerase II, Covance) or control IgG were used for immunoprecipatation. The DNA fragments were purified using a QIAquick PCR purification kit *(Qiagen*, Hilden, *Germany)*, and Q-PCR was performed using primers against appropriate promoter regions. The quantitative ChIP signals (Ct values) were normalized with respect to the input control.

### Gelatin zymography assay

Cells were seeded in 6-well plates, grown to confluence, washed three times with serum-free medium containing 0.1% bovine serum albumin (Sigma), and preincubated with serum-free medium for 3 hr. The culture medium was collected and diluted 1:1 (v/v) with 2x SDS sample buffer and heated for 30 min at 37°C. The diluted medium (50 μl) was separated on an 8% SDS-PAGE gel containing 1 mg/ml gelatin. The gel was washed twice with 2.5% Triton X-100, incubated for 15 min with developing buffer (0.2 M NaCl, 0.04 M Tris-HCl, and 0.01 M CaCl_2_, pH 7.5) and transferred to fresh developing buffer for 48 hr. The gel was fixed in fixing buffer [45% (v/v) methanol, and 10% (v/v) acetic acid], stained with 0.125% Coomassie blue, and destained for visualization of the gelatin-specific enzyme bands.

### DNA pull-down assay

DNA pull-down assays were performed as previously described [42], with some modification. The double-stranded annealed biotinylated probes (100~250 pmole) were incubated with 25 μl M-280 Streptavidin Dynabeads (Invitrogen) in recommended binding buffer for 1 hr at room temperature. In parallel, 100~250 μg nuclear extracts and salmon sperm DNA 2.5μg were incubated with 25 μl unconjugated Dynabeads (Invitrogen) 5. for 30 min at room temperature. The pre-cleared nuclear extract was incubated with the probe-bounded beads for 1 hr at room temperature. The Dynabead-bound complexes were washed six times with binding buffer containing 0.5% NP-40, and the DNA-bound proteins were eluted in SDS sample buffer and assayed by Western blotting.

### MMP-9 ELISA

Plasma samples collected from four NPC patients at different time points were diluted 40-fold with Calibrator Diluent buffer (R&D Systems, Minneapolis MN). Standards, controls and samples were added to microplate and incubated for 2 hr at room temperature. The samples were then washed three times and incubated with the substrate solution. The amount of MMP-9 in the samples was measured by ELISA reader at O.D. 450 nm, and compared with the standards.

## Supplementary Figures, Methods and Tables






