# Supplementary material for: Aberrantly hypermethylated Homeobox A2 derepresses metalloproteinase-9 through TBP and promotes invasion in Nasopharyngeal carcinoma
Source: Oncotarget. 2013 Nov 4;4(11):2154–65. doi: 10.18632/oncotarget.1367 (PMC3875777; doi:10.18632/oncotarget.1367)
Supplement: Supplementary file 1 [file oncotarget-04-2154-s001.pdf]

Aberrantly hypermethylated Homeobox A2 derepresses metalloproteinase-9 through TBP and promotes invasion in Nasopharyngeal carcinoma - li et al

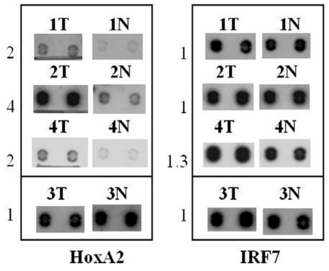

Supplementary Figure 1: Hybridized signals of HOXA2 and IRF7 in 4-paired NPC samples in TranSignal™ methylation promoter array. MBP-enriched methylated genomic DNA samples from four paired samples (NPC tumor, T; adjacent normal, N) were individually hybridized with a TranSignal™ methylation promoter array (Panomics). The hybridization signal obtained from NPC tumor versus adjacent normal (considered as control) of the *HOXA2* and *IRF7* (control) promoters are indicated in the left and right panels, respectively. The fold change of the hybridization signal of NPC tumor relative to adjacent normal was also indicated.

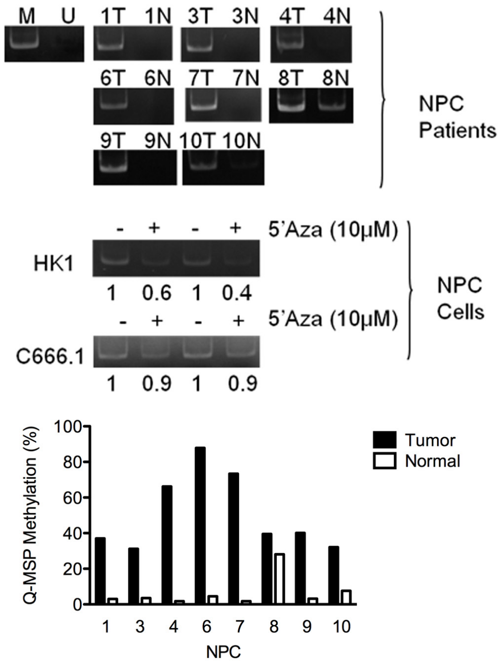

Supplementary Figure 2: Methylation specific PCR (MSP) analysis of HOXA2 promoter in NPC samples. Quantitative methylation-specific PCR (Q-MSP) analysis using "M primers" was carried out on the *HOXA2* promoter from -310~ -131 using bisulfite-treated genomic DNA of paired NPC samples (T and N) and NPC cell lines (HK1, C666.1) with or without 10 μM 5'Aza treatment. Q-MSP-amplified methylated *HOXA2* DNA was analyzed and run on the 6% acrylamide gel. The quantitative values of the Q-MSP methylation percentage of each paired NPC sample are shown in the lower panel (Tumor ■, Normal □).

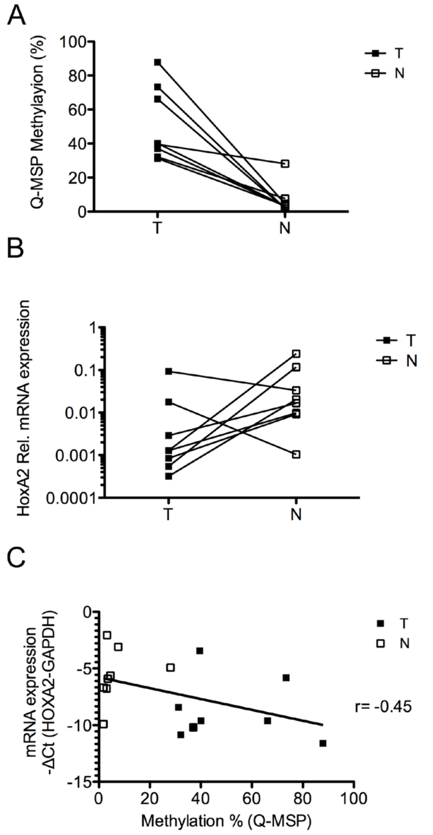

Supplementary Figure 3: HOXA2 methylation status and RNA expression were compared in eight NPC paired samples of tumor and adjacent normal tissues. (A) Comparison of Q-MSP methylation percentage (%) of eight paired NPC tumor (T) and non-tumor (N) tissues. Significant difference between T and N was found (paired T-Test, p=0.0018). (B) Comparison of the relative quantitative values of *HOXA2* mRNA level (relative to GAPDH) (-ΔCt) of eight paired NPC (T) and (N) tissues (paired T-Test, p=0.0766). (C) Moderate negative correlation between *HOXA2* DNA methylation level (Q-MSP methylation %) and the relative *HOXA2* RNA expression levels (relative to GAPDH) (-ΔCt) in NPC clinical samples; Pearson's correlation coefficient (r = -0.45) was observed.

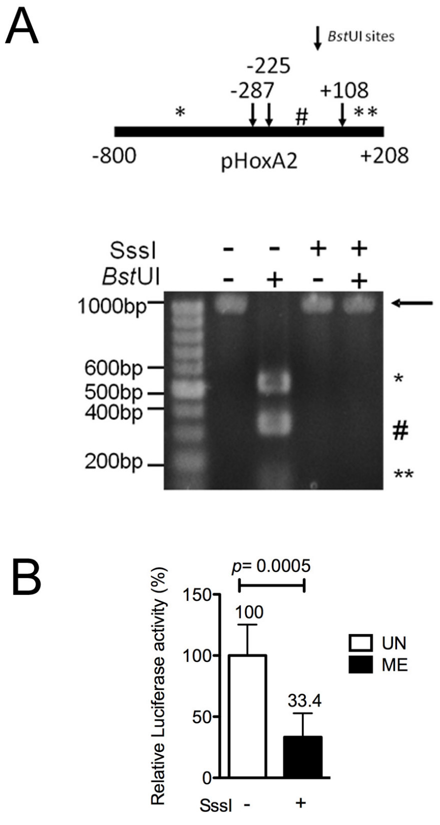

Supplementary Figure 4: Patch methylation of HOXA2 promoter. (A) The methylation efficiency of the *E. coli* methylase, SssI, on the *HOXA2* promoter (-800~ +208) was examined by digestion with a methylation-sensitive restriction enzyme. The upper panel indicates the *Bst*UI sites and the DNA fragments (* 513 bp, # 333 bp, and ** 100 bp) obtained following *Bst*UI digestion. The lower panel shows the results from 2% agarose gel electrophoresis of the *Bst*UI-digested *HOXA2* promoter fragments obtained with or without SssI treatment. (B) *HOXA2* promoter fragment (-800~ +208) was enzyme digested, recovered and treated with or without the *E. coli* methylase, SssI. Methylated or unmethylated DNA was re-ligated into pGL3 basic to generate the pGL3/pHOXA2^{me} and pGL3/pHOXA2^{un}, respectively. Unligated DNA was digested by exonuclease III. Equal amount of ligated methylated (pGL3/pHOXA2^{me}) or unmethylated (pGL3/pHOXA2^{un}) recombinant reporter DNA, and 10ng of pCMV/renilla were transfected into 293T cells. Relative promoter activity was measured by dual-luciferase reporter assays and the data were normalized with respect to renilla activity. Three independent experiments with duplicates were performed.

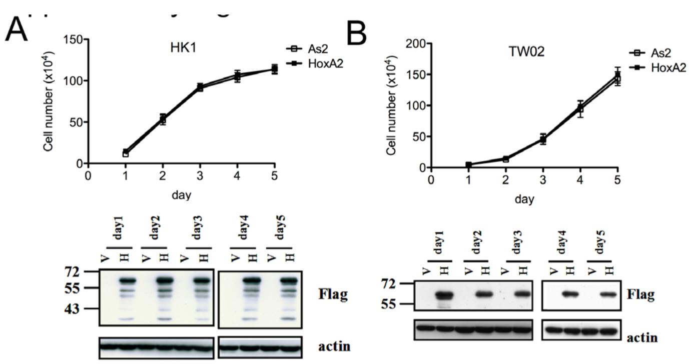

Supplementary Figure 5: Cell proliferation ability of HOXA2 in HK1 and TW02 cells. The cell proliferation abilities of (A) HK1 cells stably expressing HOXA2 and (B) TW02 cells stably expressing HOXA2 were analyzed. Cell numbers over 5 days are plotted as mean±SD. Western blotting was used to confirm the expression of exogenous HOXA2 (anti-Flag) and the internal control (anti-actin).

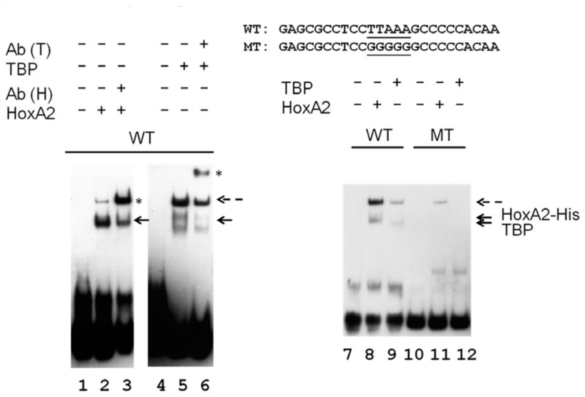

Supplementary Figure 6: EMSA analysis of HOXA2 and TBP binding affinity toward MMP-9 promoter. EMSA was used to analyze the binding affinity of purified recombinant HOXA2 and TBP for biotinylated probes representing wild-type and mutated versions of the *MMP-9* TATA box. Arrows and dotted arrows indicate the DNA-protein and higher molecular weight DNA-protein complexes, respectively. Antibodies against HOXA2 or TBP were used for supershift assays.

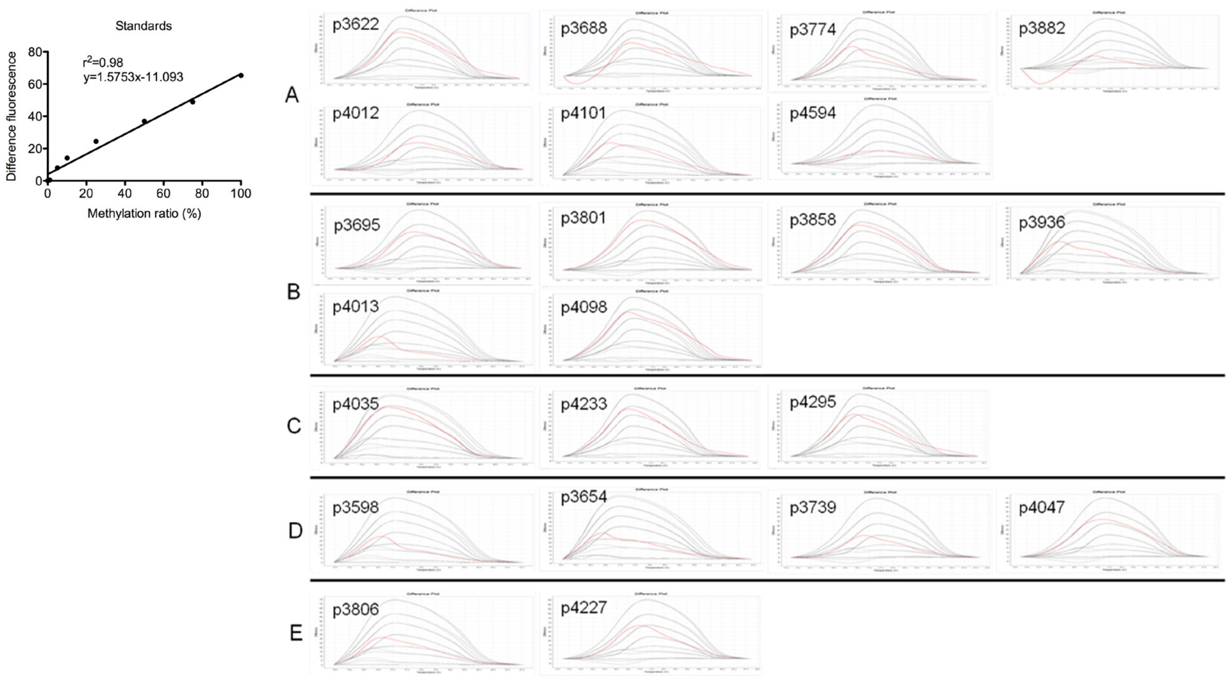

Supplementary Figure 7. HRM analysis of HOXA2 methylation status from 22 NPC samples. Each difference plot analyzed by HRM software v2.0 (Life Technology) indicates the *HOXA2* methylation status (red) of NPC patients' plasma compared with different combination of unmethylated (0%) and methylated (100%) standard plasmids (0%, 25%, 50%, 75% and 100% methylation, grey lines). The difference fluorescence values of the *HOXA2* amplicons from the standards and the plasma samples corresponding to the Tm (78.5°C) were measured from the HRM difference plot. Linear regression trend line of the standards and the equation of difference fluorescence versus methylation % were obtained. Methylation % of *HOXA2* in each sample was calculated according to the trend line equation.
