# Supplementary material for: Aberrantly hypermethylated Homeobox A2 derepresses metalloproteinase-9 through TBP and promotes invasion in Nasopharyngeal carcinoma
Source: Oncotarget. 2013 Nov 4;4(11):2154–65. doi: 10.18632/oncotarget.1367 (PMC3875777; doi:10.18632/oncotarget.1367)
Supplement: Supplementary file 2 [file oncotarget-04-2154-s002.pdf]

Supplementary Table 1. Primers sequence for PCR.

Primers		
Primer	Sequence (Forward and Reverse)	
Bisulfite sequencing-HOXA2	F	TTTTAATAGAATTTATGTGGTTGGGA
	R	ATAAAAACAAAATTAAAATTAAACC
Methylation-specific PCR-HOXA2	F	TTTTCGATAGTTTTAAATAATGCGC
	R	ACGTAAAAACGAAAATACCTACGTA
High resolution melting-HOXA2	F	TTAGATTGAGGTGTTTAAATG
	R	GATAGATTTTTGTAGATTAG
QRT-PCR-HOXA2	F	ACAGCGAAGGGAAATGTAAAAGC
	R	GGGCCCCAGAGACGCTAA
QRT-PCR-Actin	F	CGGGAAATCGTGCGTGACATTAAG
	R	TGATCTCCTTCTGCATCCTGTCGG
QRT-PCR-MMP9	F	CACTGTCCACCCCTCAGAGC
	R	GCCACTTGTCGGCGATAAGG
cDNA-HOXA2	F	GAGGCTAGCATGAATTACGAATTTGAG
	R	CGAATTCCTATTTATCGTCATCATCTTTGTAGTC
HOXA2 Promoter deletion constructs :		
(-435)	F	GCTAGATCTGAACTTATGTGGCTGGGACGC
(-171)	F	GATAGATCTTTGCAGATCAGGAGGCACGCAGGC
(-110)	F	CCCAGATCTCACCAACATATATGTATTTTTGC
(+208)	R	CCCAAGCTTTTAGCCTAGGAAAAAGGCGAGCGCAGAGG
MMP9 Promoter deletion constructs:		
(-1650)	F	AGTAGATCTTTCGAAACCAGCCTGGTCAACGTA
(-1150)	F	TAAAGATCTAAAGGGGAAGGCATTTACTCCAGGT
(-1073)	F	AGTAGATCTCATGGTCTTTTGGCAGGGTCTCGCT
(-703)	F	GTCAGATCTGAAGAGAGTAAAGCCATGTCTGC
(-205)	F	TTGAGATCTTTGGCAGTGGAGACTGCGGGCAGT
(+150)	R	ACCAAGCTTAGCTGCCTGTCGGTGAGATTGGTTC
ChIP-HOXA2 p300-1	F	GATAGATCTTTGCAGATCAGGAGGCACGCAGGC
	R	GCCAGCGAGGCTTTATTTACACTT
p300-2	F	GCTAGATCTGAACTTATGTGGCTGGGACGC
	R	CGGTGGCTGCCCTCTGCCTC
Control	F	CCCAGATCTCACCAACATATATGTATTTTTGC
	R	CCCAAGCTTTTAGCCTAGGAAAAAGGCGAGCGCAGAGG
ChIP-MMP9 TTAAA	F	TGAGTCAGCACTTGCCTGTC
	R	ATGGTGAGGGCAGAGGTGTC

EBV W repeat	F	CCCAACACTCCACCACACC
	R	TCTTAGGAGCTGTCCGAGGG

Supplementary Table 2. Sequence for DNA pull-down and EMSA biotinylated primers

5'-Biotinylated Oligonucleotides		
Probe	Sequence (Forward and Reverse)	
HOXA2 p300-1 WT	F	CCCCCACGTACTCCGGG
	R	CCCGGAGTACGTGGGGG
ME	F	CCCCCA^{m}CGTACTC^{m}CGGG
	R	CC^{m}CGGAGTA^{m}CGTGGGGG
MT	F	CCCCCAAAAAAAAAGGG
	R	CCCTTTTTTTTTGGGGG
3 copies of p300 consensus	F	GGGAGTGGGGAGTGGGGAGTGAAA
	R	TTTCACTCCCCACTCCCCACTCCC
MMP9 TATA WT	F	GAGCGCCTCCTTAAAGCCCCCACAA
	R	TTGTGGGGGCTTTAAGGAGGCGCTC
MT	F	GAGCGCCTCCGGGGGGCCCCCACAA
	R	TTGTGGGGGCCCCCCGGAGGCGCTC
MMP9 control	F	AAAAATAAAATAAAATAAATAAATA
	R	TATTTATTTATTTTATTTTATTTTT

Supplementary Table 3. List of the over-expressed eight out of twenty-two MMPs (T/N >1.5-fold) from the cDNA microarray (Affymetrix) of 9 NPC tumors versus one combined adjacent normal

**Genes**	**Probes**	**RNA-fold (T/N)**	***p*-value**
MMP9	203936_s_at	40.46	0.003
MMP12	204580_s_at	27.77	0.003
MMP3	205828_s_at	14.2	0.059
MMP11	203878_s_at	4.708	0.202
MMP13	205959_s_at	4.293	0.42
MMP28	219909_s_at	2.911	0.251
MMP19	204575_s_at	2.625	0.09
MMP14	160020_s_at	1.806	0.013

Supplementary Table 4. EBV copy number, HOXA2 methylation percentage of cell-free DNA and MMP9 protein amount detected from five NPC patients' plasma collected at different time.

Patient	EBV copies/ml	EBV copies (Log)	HOXA2 methylation (%)	MMP-9 conc. (ng/ml)
p3622	4178	3.62	63.6	56.11
p3688	215853	5.33	58	32.15
p3774	89150	4.95	26	53.85
p3882	171	2.23	21.4	N.D.
p4012	2024	3.31	47.1	54.73
p4101	3215	3.51	34.9	27.75
p4594	2904	3.46	19.5	33.98
p3695	9167	3.96	58.6	47.91
p3801	54098780	7.73	74.4	109.50
p3858	16025908	7.20	63.6	92.55
p3936	73749	4.87	25.9	60.79
p4013	13895	4.14	4.4	44.81
p4098	547169	5.74	65.5	108.58
p4035	5545203	6.74	67.6	24.07
p4233	133593	5.13	58	57.35
p4295	5595	3.75	43.6	6.56
p3598	81	1.91	11.6	12.72
p3654	52	1.72	18.2	16.04
p3739	872	2.94	27	18.28
p4047	24342688	7.39	59	24.10
p3806	89787	4.95	26.5	20.69
p4277	134109	5.13	50.4	67.06

N.D.: Non-detectable.
