# Supplementary material for: Aberrantly hypermethylated Homeobox A2 derepresses metalloproteinase-9 through TBP and promotes invasion in Nasopharyngeal carcinoma
Source: Oncotarget. 2013 Nov 4;4(11):2154–65. doi: 10.18632/oncotarget.1367 (PMC3875777; doi:10.18632/oncotarget.1367)
Supplement: Supplementary file 3 [file oncotarget-04-2154-s003.pdf]

Aberrantly hypermethylated Homeobox A2 derepresses metalloproteinase-9 through TBP and promotes invasion in Nasopharyngeal carcinoma - li et al

Supplementary Information

Materials and Methods

Promoter methylation array hybridization

Differential methylation hybridization was performed using the TranSignal Promoter Methylation Array (Panomics, Fremont, CA) according to the manufacturer's recommendations. Briefly, 5 μg of genomic DNA from four NPC biopsy/adjacent tissues pairs was MseI digested, purified and ligated with linkers. To enrich for methylated DNA, linker-adapted genomic DNA was affinity purified using methyl binding protein (MBP) columns. PCR amplification was used to label the eluted methylated DNA with biotin-dCTP, and the biotinylated PCR products were hybridized to the methylation promoter array. The hybridization signals, which were proportional to the amount of methylated DNA in the sample, were detected with a streptavidin-horse radish peroxidase (HRP)-based developing system. Genes that showed higher signals in tumor samples compared with the paired adjacent normal samples in least three out of four NPC cases were identified as putative differentially methylated genes.

Quantitative methylation-specific PCR (Q-MSP)

Bisulfite-converted DNA (20 ng) was subjected to real-time Q-MSP on an iCycler (Bio-Rad, Hercules, CA). Methylation levels were determined by comparing the threshold cycle (Ct) number to a standard curve generated using different amounts of Universal Methylated DNA (Millipore, Billerica, MA) as the template. A CpG-free region of the actin gene was used as an internal control. To determine the relative methylation level for each sample, the methylation ratio between the target gene and the internal reference gene was multiplied by 100 to yield a percentage.

RNA extraction and quantitative real-time PCR

Total RNA was extracted and DNaseI treated. Reverse transcription (RT) was performed using Improm-II reverse transcriptase (Promega, Madison, WI) according to the manufacturer's protocol. Q-RT-PCR was performed using SYBR master mixture (Kapa Biosystems, Woburn, MA) on an IQ5 system (Bio-Rad), according to the manufacturer's instructions. The relative gene expression level was determined with respect to the internal controls, GAPDH or β-actin, and calculated by the $2^{-\Delta Ct}$ method. Three independent experiments were performed, each in duplicate.

Construction of HOXA2 expression vectors

RNA from human peripheral blood cells was reverse transcribed to cDNA. The HOXA2 cDNA was PCR-amplified and subcloned into the *Bam*HI and *Hind*III sites of the pCMV-3tag-8 vector (Stratagene, La Jolla, CA) to generate the Flag-tagged expression clone, pCMV/HOXA2. The Flag-tagged HOXA2 cDNA was PCR-amplified and subcloned into the *Nhe*I and *Eco*RI sites of the lentivirus-based vector, pLKO_AS2.neo (National RNAi Core Facility, NRCF, Academic Sinica, Taiwan) to generate the Flag-tagged lentivirus expression clone, pLKO/HOXA2. The sequences of all constructs were confirmed.

Recombinant lentivirus preparation and selection of stable cells

The expression vector, pLKO/HOXA2, or vector control, pLKO_AS2.neo; the envelope plasmid, pMD2.G; and the packaging plasmid, pCMV-dR8.91; were all co-transfected into 293FT producer cells by the calcium phosphate method, as recommended in the NRCF protocol (http://rnai.genmed.sinica.edu.tw). Media containing recombinant lentiviruses (expressing HOXA2 or vector) were collected 48 hr post-transfection and filtered. HK1 or TW02 cells (30~40% confluence) were infected by recombinant lentivirus supplemented with 8 μg/ml polybrene (Sigma-Aldrich, St. Louis, MO). Twenty-four hr post-infection, the medium was replaced with fresh complete medium containing 500 μg/ml G418 for selection of stably infected cells.

In vitro patch methylation

A *HOXA2* promoter fragment (-800~ +200) was digested from pHOXA2/pGL3 and purified, and 1 μg of the fragment was *in vitro* methylated using the SssI methylase (NEB, Ipswich, MA) according to the manufacturer's recommendations. The methylation status of pHOXA2 was confirmed using the methylation-sensitive restriction enzyme, *Bst*UI (NEB). The methylated or unmethylated DNA fragments were re-ligated (i.e., "patched") into the pGL3-basic vector to generate pHOXA2me/pGL3 and pHOXA2un/pGL3, respectively. Unligated DNA was digested with exonuclease V, and ligation efficiency was monitored by assessing the transformation efficiency in *E. coli*. The patched methylated or unmethylated reporters were individually transfected into 293T cells for analysis of promoter activity.

Electrophoretic mobility shift assay (EMSA)

EMSA was performed using a LightShift® Chemiluminescent EMSA Kit (Thermo Scientific, Waltham, MA). Wild-type or mutant complementary probes 5' end-labeled with biotin were synthesized and annealed by heating at 95^0C for 10 min followed by a gradual cooling to room temperature. The annealed biotin-labeled probes (20 fmol) and recombinant protein were incubated at 37°C for 20 min, and then cold competitor DNA duplexes were added at a 50~200-fold molar excess. DNA-protein complexes were separated on 6% 1X TBE non-denaturing polyacrylamide gels and transferred to nylon membranes (PerkinElmer, Waltham, MA). The membranes were cross-linked by the application of 120 mJ/cm^2 of UV, and the results were detected using a Chemiluminescent Nucleic Acid Detection Module (Thermo Scientific), according to the manufacturer's instructions.

High-resolution melting analysis of plasma DNA from NPC patients

Plasma samples were obtained from NPC patients, and cell-free genomic DNA was extracted using a QIAamp® DNA Mini and Blood Mini kit (Qiagen). The cell-free genomic DNA was bisulfite converted using an EZ DNA methylation kit (Zymo, Irvine CA) and amplified using SYTO9 saturated dye (Life Technologies) and Kapa Taq polymerase in an ABI-7500 Fast PCR System (Life Technologies). The results were analyzed using the High Resolution Melting (HRM) software v2.0 (Life Technologies).
